# 1697. Dose-response Studies of the Novel Bacterial Leucyl-tRNA Synthetase Inhibitor, Epetraborole, in the Intracellular Hollow Fiber System Model of *Mycobacterium avium complex* Lung Disease

**DOI:** 10.1093/ofid/ofac492.1327

**Published:** 2022-12-15

**Authors:** Moti Chapagain, Shruti Athale, Jotam Pasipanodya, David Howe, M R K Alley, Tawanda Gumbo

**Affiliations:** Praedicare Inc., Dallas, Texas; Praedicare Inc., Dallas, Texas; Praedicare Inc., Dallas, Texas; Praedicare Inc., Dallas, Texas; AN2 Therapeutics, Menlo Park, California; Praedicare Inc., Dallas, Texas

## Abstract

**Background:**

Based on meta-analyses the current standard-of-care regimen (SOC) for *Mycobacterium avium* complex (MAC) lung disease achieves a sustained sputum conversion rate of ∼54% at best. Epetraborole (EBO) is a boron-containing oral inhibitor of bacterial leucyl-tRNA synthetase, an essential enzyme in protein synthesis; EBO demonstrates potent activity against nontuberculous mycobacteria. To identify EBO exposure-effect parameters we used the intracellular hollow fiber system model of intracellular pulmonary MAC (HFS-MAC).

**Methods:**

EBO was administered once daily at 8 different doses to HFS-MAC replicates for 28 days to achieve a half-life (t½) of 10.4h and the 0-24h area under the concentration-time curves (AUC_0-24_) that cover the observed AUC values in humans. The SOC combination of clarithromycin (CLR), ethambutol (EMB), and rifabutin (RFB) at human intrapulmonary pharmacokinetic (PK) concentrations was used. The central compartment of each HFS-MAC unit was sampled throughout the 28 days to assess PK parameters as was the peripheral compartment for total bacterial burden and the EBO-resistant bacterial burden. EBO AUC versus total MAC burden was modeled using the inhibitory sigmoid maximal effect (E_max_) model. EBO AUC versus EBO-resistant MAC burden was modeled using a quadratic function.

**Results:**

Measured EBO concentrations demonstrated a t_1/2_ of 10h. For SOC, the AUC for CLR, EMB, and RFB was 60 mg*h/L (t_1/2_=6h), 39 mg*h/L (t_1/2_=8h), and 1.5 mg*h/L (t_1/2_=45h), respectively, similar to human lung concentrations. Changes in MAC burden over 28 days (**Fig. 1**) show that highest EBO exposures matched SOC until day 14. The exposure versus effect on each sampling day is shown in **Fig. 2**. The EBO AUC mediating 50% of E_max_ (EC_50_) was an AUC of 22 mg*h/L (95% confidence interval [CI]: 16-70), and the EC_80_ was an AUC of 47.5 mg*h/L (CI: 34.6-151.2). The relationship between AUC and EBO-resistant subpopulation (**Fig. 3**) shows that an AUC_0-24_ of 47.5 mg*h/L, the same as the EC_80_ for microbial kill, was associated with resistance suppression, as was the addition of SOC to EBO.

**Figure 1.**
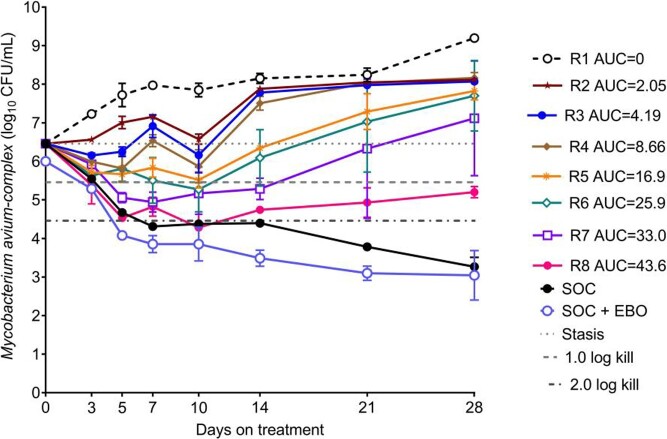
Changes in bacterial burden over the 28-day dosing period.

Bacterial burden on serial samples from all HFS-MAC units and replicates over 28days are shown. The highest epetraborole exposures demonstrated the same effectiveness as the three drug SOC until day 14, after which it failed as resistance emerged. On the other hand adding epetraborole at an AUC of 43.6 mg*h/L to the SOC led to much faster kill (i.e, higher kill slopes) than SOC or the epetraborole monotherapy at the same AUC.

**Figure 2.**
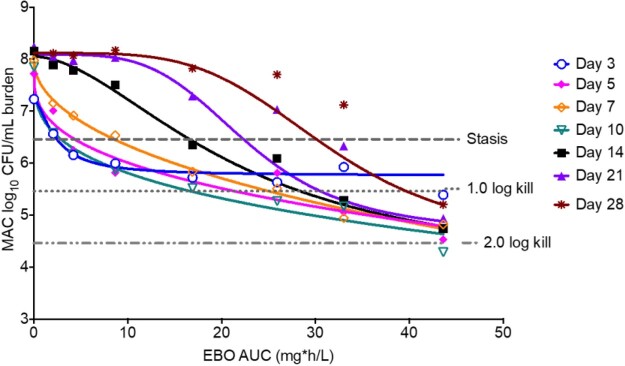
Epetraborole exposure versus effect on bacterial burden.

Shown are inhibitory sigmoid Emax curves for epetraborole AUC versus log10 CFU/mL on each sampling day, as well as various extents of microbial kill such as stasis (holding burden similar to day 0), 1 log CFU/mL kill and 2 log CFU/mL kill. Beyond day 3, epetraborole killed > 1log CFU/mL, which means it was rapidly bactericidal.

**Figure 3.**
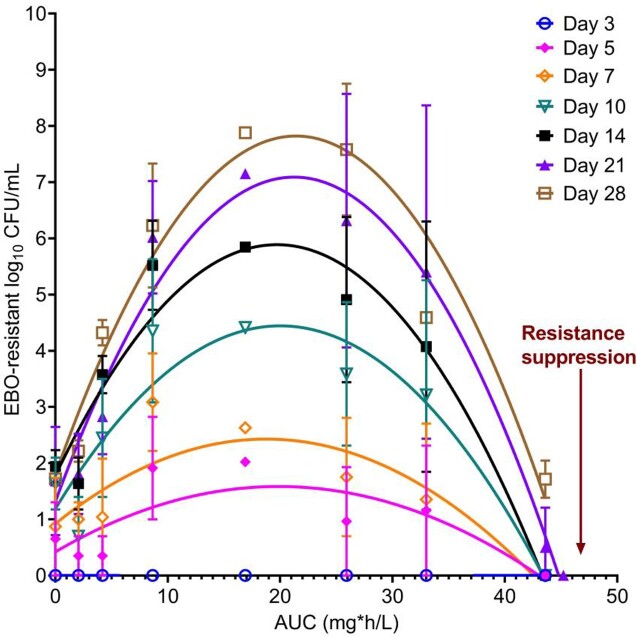
Epetraborole exposure versus epetraborole-resistant subpopulation.

Drug-resistant burden is shown as log10 CFU/mL. The addition of SOC to EBO totally suppressed emergence of any resistance on all days.

**Conclusion:**

EBO monotherapy with an AUC_0-24_ > 16.9 mg*h/L killed > 1.0 log_10_ CFU/mL of MAC compared to day 0. At the EC_80_, EBO killed at least 2.0 log_10_ CFU/mL, thus was highly bactericidal. EBO plus SOC demonstrated resistance suppression.

**Disclosures:**

**Moti Chapagain, MD, PhD**, Praedicare Inc.: Stocks/Bonds **Shruti Athale, PhD**, Praedicare Inc.: Stocks/Bonds **Jotam Pasipanodya, MD, PhD**, Praedicare Inc.: Employee **MRK Alley, PhD**, ABBOTT LABS: Stocks/Bonds|ABBVIE: Stocks/Bonds|AN2 Therapeutics: Author on epetraborole patent|AN2 Therapeutics: Salary|AN2 Therapeutics: Ownership Interest|AVANOS MED INC: Stocks/Bonds|NABRIVA THERAPEUTICS PLC: Stocks/Bonds|NOVARTIS AG: Stocks/Bonds **Tawanda Gumbo, MD**, Hydronium Biopharma: Ownership Interest|Hydronium Biopharma: Stocks/Bonds|Praedicare Africa Pvt Ltd: Board Member|Praedicare Africa Pvt Ltd: Ownership Interest|Praedicare Africa Pvt Ltd: Stocks/Bonds|Praedicare Inc.: Patents|Praedicare Inc.: Ownership Interest|Praedicare Inc.: Stocks/Bonds.

